# Work, Sleep, and Play

**Published:** 1905-04-22

**Authors:** 


### Work, Sleep, and Play.

The paper read at the recent Conference on School Hygiene by Dr. Clement Dukes, and published in the current number of the "Journal of the Sanitary Institute," is worthy of attention, not only from teachers, but also from parents, for it deals with the subject of the amount of sleep required by children, and lays down, with the authority of the author's large experience, principles which are certainly quite as often neglected or ignored at home as at school, especially among the poorer classes of the community, whose children are constantly deprived of natural rest by being dragged about in connection with the pleasures or the "outings" of the parents. Dr. Dukes points out that in primary schools children of three years of age pass the same number of hours in school as those of 14 years of age, and that in secondary schools the child of 14 has the same hours of work allotted to him as the youth of nineteen. He declares that at the beginning of the day, after a night's rest, at the beginning of a week, after the Saturday and Sunday rest, and at the beginning of a term, after the rest of the vacation, the best work is always accomplished, and quotes Archbishop Temple and Dr. Arnold in support of the proposition "that within a fortnight or three weeks of the resumption of school, the boys who had followed their desires, and simply read when it pleased them so to do, had immeasureably surpassed those who had been studying all through the vacation."

Dr. Dukes has not been content with laying down these general principles, but has further sought to embody them in simple rules capable of universal application. He reminds us that an infant, when fed from the natural source, sleeps, in the first four months of its life, about twenty out of the twenty-four hours; and that, if we follow nature during the years of formation and development, adequate sleep must not only be permitted, but enforced. He asserts, moreover, that the deleterious effects upon the growing brain of excessive hours of work during school life cannot be compensated for by an extra amount of sleep, since Nature repudiates the attempt, and will not permit a forced brain to rest. The long hours of work demanded of the younger children, with the short time allowed for sleep, could not be continued if the scheme were in force all the year round, as there would be no children left at school. It is only the vacations that prevent a complete breakdown. He quotes Dr. Clouston as an authority for the assertion that, during the last thirty years, *i.e.* since the coming into force of the Education Act of 1870, there has been a decreasing amount of staying power, on the part of the brain, to the extent of about 40 per cent., and he called upon his hearers to consider what share in this "terrible fact" must be attributed to schools, with the excessive hours of work which they demand from the early years of childhood, and with the inadequate amount of sleep which these demands permit. His view of the case would not suffer the children who, at the age of five, pass from the infants' to the primary school, to work at lessons for more than a single hour out of the twenty-four, and would secure to them thirteen hours and a-half of sleep. Between the ages of five and fourteen, the hours of work may be gradually extended to five, and the hours of sleep as gradually diminished to ten and a-half. In the secondary school he would permit six hours of work from fourteen to sixteen, seven hours from sixteen to eighteen, and eight from eighteen to nineteen, with corresponding reductions of sleep to ten hours, nine and a-half, and nine; while at the University, and up to the age of twenty-three, although the work should not be suffered to exceed eight hours, the sleep may be brought down to the same duration. If we may assume that Dr. Dukes is correct in his view of the rest requirements of the child, and of his capacity, even when untired, of employing his brain in intellectual labour, and if we then turn to recent official disclosures concerning the manner in which thousands of school children are kept at employment in various forms of industry before the school hours begin or after they have terminated, we shall not have to look much farther for at least one serious cause of physical degeneration among the individuals of the coming race. We shall see, only too plainly, that our "educational" legislation has not only failed to be in accordance with physiology, but that, in many highly important respects, it has been framed in direct violation of physiological principles.

